# Type and Gene Location of *KIT* Mutations Predict Progression-Free Survival to First-Line Imatinib in Gastrointestinal Stromal Tumors: A Look into the Exon

**DOI:** 10.3390/cancers13050993

**Published:** 2021-02-27

**Authors:** Lorena Incorvaia, Daniele Fanale, Bruno Vincenzi, Ida De Luca, Tommaso Vincenzo Bartolotta, Roberto Cannella, Gianni Pantuso, Daniela Cabibi, Antonio Russo, Viviana Bazan, Giuseppe Badalamenti

**Affiliations:** 1 Department of Biomedicine, Neuroscience and Advanced Diagnostics (Bi.N.D.), Section of Medical Oncology, University of Palermo, 90127 Palermo, Italy; lorena.incorvaia@unipa.it (L.I.); viviana.bazan@unipa.it (V.B.); 2 Department of Surgical, Oncological and Oral Sciences, Section of Medical Oncology, University of Palermo, 90127 Palermo, Italy; fandan@libero.it (D.F.); ida.deluca@unipa.it (I.D.L.); giuseppe.badalamenti@unipa.it (G.B.); 3 Department of Medical Oncology, Biomedical Campus, University of Rome, 00128 Rome, Italy; b.vincenzi@unicampus.it; 4 Department of Biomedicine, Neuroscience and Advanced Diagnostics (Bi.N.D.), Section of Radiology, University of Palermo, 90127 Palermo, Italy; tommasovincenzo.bartolotta@unipa.it (T.V.B.); roberto.cannella@you.unipa.it (R.C.); 5 Department of Surgical, Oncological and Oral Sciences, Section of General and Oncological Surgery, University of Palermo, 90127 Palermo, Italy; gianni.pantuso@unipa.it; 6 Department of Health Promotion, Mother and Child Care, Internal Medicine and Medical Specialties, Pathologic Anatomy Unit, University of Palermo, 90127 Palermo, Italy; daniela.cabibi@unipa.it

**Keywords:** gastrointestinal stromal tumors, GIST, imatinib, KIT, mutations, predictive biomarkers

## Abstract

**Simple Summary:**

Although effective in the majority of patients, the progression-free survival (PFS) to imatinib treatment can vary widely in effectiveness. Based on the known predictive role of tyrosine kinase (*KIT*) and platelet-derived growth factor receptor α (*PDGRA*) tumor genotypes, the differential clinical response to first-line imatinib treatment might be related to the different types and gene locations of the mutations. In our study, metastatic patients with gastrointestinal stromal tumors (GIST)-carrying *KIT* exon 11 deletion or a deletion/insertion involving codons 557/558 showed significantly shorter PFS to imatinib compared with those with deletion in codons other than 557/558 and patients with exon 11 duplication, insertion or single nucleotide variants (SNVs). Conversely, the latter subgroup showed the longest PFS first-line to imatinib. These results highlight the predictive role of pathogenic variant (PV) type and codon location in GIST, and can support stratification via mutational status in future clinical trials.

**Abstract:**

In previous studies on localized GISTs, *KIT* exon 11 deletions and mutations involving codons 557/558 showed an adverse prognostic influence on recurrence-free survival. In the metastatic setting, there are limited data on how mutation type and codon location might contribute to progression-free survival (PFS) variability to first-line imatinib treatment. We analyzed the type and gene location of *KIT* and *PDGFRA* mutations for 206 patients from a GIST System database prospectively collected at an Italian reference center between January 2005 and September 2020. By describing the mutational landscape, we focused on clinicopathological characteristics according to the critical mutations and investigated the predictive role of type and gene location of the *KIT* exon 11 mutations in metastatic patients treated with first-line imatinib. Our data showed a predictive impact of *KIT* exon 11 pathogenic variant on PFS to imatinib treatment: patients with deletion or insertion/deletion (delins) in 557/558 codons had a shorter PFS (median PFS: 24 months) compared to the patients with a deletion in other codons, or duplication/insertion/SNV (median PFS: 43 and 49 months, respectively) (*p* < 0.001). These results reached an independent value in the multivariate model, which showed that the absence of exon 11 deletions or delins 557/558, the female gender, primitive tumor diameter (≤5 cm) and polymorphonuclear leucocytosis (>7.5 109/L) were significant prognostic factors for longer PFS. Analysis of the predictive role of *PDGFRA* PVs showed no significant results. Our results also confirm the aggressive biology of 557/558 deletions/delins in the metastatic setting and allow for prediction at the baseline which GIST patients would develop resistance to first-line imatinib treatment earlier.

## 1. Introduction

The discovery of constitutive activating mutations in the proto-oncogene receptor tyrosine kinase (*KIT*) or platelet-derived growth factor receptor α (*PDGFRA*) oncogenes as molecular drivers of most gastrointestinal stromal tumors (GISTs) [1,2] has radically changed our knowledge on their tumor biology. Activating mutations in *KIT* (70–80% of GISTs) or *PDGFRA* (5–10% of GISTs) disrupt the normal autoinhibitory state of receptor tyrosine kinase (RTK), leading to the constitutive, ligand-independent activation of Ras/Raf/MAPK, JAK/STAT3 and PI3K/Akt/mTOR downstream pathways, ultimately resulting in increased cell proliferation [3,4]. The resulting aberrant receptor tyrosine kinases, known to be clinically excellent therapeutic targets, have transformed GISTs into a model for successful molecular targeted therapy [5,6]. Indeed, *KIT* and *PDGRFA* represent not only key diagnostic markers but, more importantly, they also present prognostic and predictive significance, providing a powerful tool for the therapy selection process [7,8,9,10,11]. Tumor mutational status is particularly important because GIST patients show great variability in response to medical treatment, depending on the mutation’s location [12]. GISTs with *KIT* exon 11 mutations are the most responsive to standard first-line imatinib 400 treatment, with a response rate of 80% [13], whereas *KIT* exon 9 mutations are associated with a lower response rate of 45%. These patients benefit from the higher dose of 800 mg/die [13,14]. Although most GISTs with *KIT* exon 11 mutations are highly sensitive to front-line imatinib, progression-free survival (PFS) can vary widely in this subset of patients [15,16,17]. In previous studies on localized GISTs treated with surgery and adjuvant imatinib, different types of *KIT* exon 11 mutations (mainly deletions or mutations that involve codons 557 and/or 558) showed an adverse prognostic influence on recurrence-free survival (RFS) [18,19,20]. Indeed, there are limited data on how mutation type and codon location might contribute to clinical behavior and the variability of response to therapy in metastatic GISTs treated with first-line imatinib. A previous study investigated the predictive role of *KIT* exon 11 mutations in a cohort of advanced patients, with a primary interest in pathogenic variant (PV) location. This showed that patients with alterations affecting codons 557/558 developed secondary resistance more rapidly compared to patients carrying the most distal exon 11 mutations (codon 559 or downstream) [21]. However, the potential predictive impact of specific PV type needs further elucidation in the metastatic setting.

Based on this rationale, we focused on the clinicopathological characteristics according to critical mutations by describing the mutational landscape in a broad cohort of GIST patients and investigated the predictive role of the type and codon location of the *KIT* exon 11 mutations in metastatic GIST patients treated with first-line imatinib. 

## 2. Results

### 2.1. Pathogenic Variant Classification of GIST Patients

**Figure 1:**
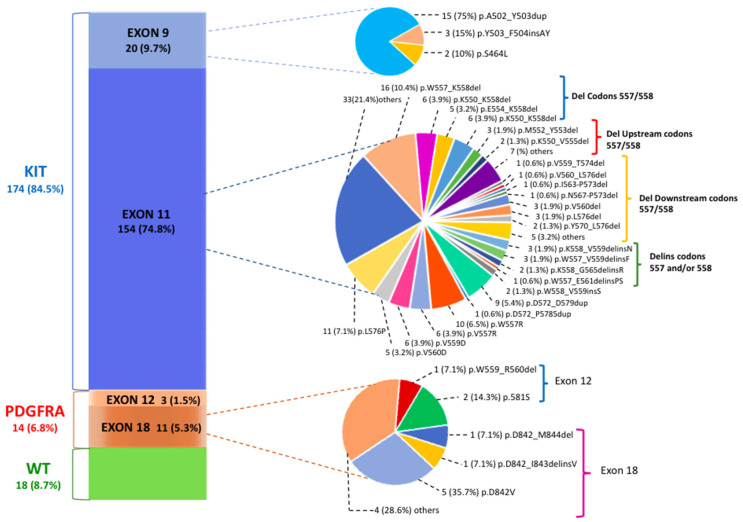
The mutational landscape of gastrointestinal stromal tumor (GIST) patients according to pathogenic variant (PV) gene location. The most common tyrosine kinase (*KIT*) exon 11 PVs are classified into deletions involving codons 557/558, deletions upstream of codons 557/558, deletions downstream of codons 557/558 and deletions/insertions affecting codons 557 and/or 558.

PV classification was assessed for 206 GIST patients; in Figure 1, we describe the genetic landscape of GIST patients according to exact PV type and codon location.

Of 206 patients, 188 showed PV mutually exclusive of *KIT* (174 patients, 84.5%) or *PDGFRA* (14 patients, 6.8%) genes. In the group of 154 patients with *KIT* exon 11 mutations (74.8%), 95 deletions (del) (61.7%), 38 single nucleotide variants (SNVs) (24.7%), 10 duplications (6.5%), 9 insertions/deletions (delins) (5.8%) and 2 insertions (1.3%) were identified. The most common site of *KIT* exon 11 mutations included codons 550–560. Sixty out of 154 (39%) patients carried deletions that spanned the critical codons 557 and/or 558. These deletions were lost either as specific isolated 557/558 deletions, such as the most common W557_K558del (n.16 patients; 7.8% of all GIST patients), or as part of larger deletions that included mutation positions upstream of codon 557 (p.K550_K558del; p.K550_E554del) and downstream of codon 558. Among the latter are included several deletions involving codons 568–570, which are located in the distal part of the exon and which seem to have an important and specific effect on KIT signaling pathways [22]. These PVs, however, are characterized by a low frequency (with less than three cases for each PV). Among the other types of *KIT* exon 11 PVs, SNVs mainly affecting codons 557–560 were particularly frequent. The p.W557R, which leads to a missense substitution in position 557 of the KIT protein with the substitution of tryptophan (W) with arginine (R), was found in 10 GIST patients (4.9%). This variant, also known as c.1669T>C, results in a single nucleotide change with the substitution of a thymine fora cytosine in position 1669 of the coding sequence of the gene.

In *KIT* exon 9, which encodes for the extracellular domain, the most common PV identified was the A502_Y503 duplication (n. 15 patients; 75% of exon 9 PVs; 7.3% of all GIST patients), which mimics the conformational change that the extracellular KIT receptor undergoes upon ligand bounding.

Concerning the *PDGFRA* gene, most mutations were identified in exon 18 (n.11 patients; 78.6% of *PDGFRA* PVs; 5.3% of the total GIST patients). The most prevalent PV was the known imatinib-resistant p.D842V (n.5 patients, 1.9% of all patients; 45.4% of all exon 18 *PDGFRA* mutated GISTs), a SNV involving the second kinase domain and consisting of a single substitution at position 842 in the A-loop of an aspartic acid (D) by a valine (V). Rarely, in-frame deletions (p.D842_M844del) or deletions/insertions (p.D842_I843delinsV) of different lengths or other SNVs (p.N848K) were detected. Finally, the *PDGFRA* exon 12 PVs 3 (1.5%) were equally rare, with one deletion (p.W559_R560del) and two SNVs (p.P581S) observed. The detailed number of patients for each mutation type and the description of the corresponding pathogenic variants are included in Table 1.

### 2.2. Metastatic GIST Patients

#### 2.2.1. Clinicopathological Characteristics According to Critical Mutations

In the group of 80 metastatic patients, 60 (75%) harbored a *KIT* exon 11 PV and 10 (12.5%) a *KIT* exon 9 PV, while *PDGRFA* mutations were found in 7 patients (8.7%) and *KIT/PDGFRA* wild-type genotypes were found in 3 patients (3.8%). Among the *KIT* exon 11 mutated patients, 25 (31.3%) showed exon 11 deletions or delins involving 557 and/or 558 codons and 35 patients (43.7%) had a deletion in codons other than 557/558 or other PV types (duplication, insertion or SNV). 

The patients with GIST who carried deletions or delins in codons 557/558 were predominantly male (64%), with gastric GISTs (60%), a primitive tumor diameter of >5 cm (92%) and a mitotic index of >5/50 HPF (76%), without predominant hepatic (40%) or peritoneal (44%) metastatic involvement. Patients with GIST who carried *KIT* exon 11 PVs not including 557/558 deletions or delins showed tumors that most frequently originated in the small bowel (site of origin, small bowel 51.5% vs. stomach 22.8%), most frequently had large primitive tumors (diameter > 5 cm 77.2% vs. diameter ≤ 5 cm 22.8% of patients) and most frequently had a high median mitotic rate (mitosis >5/50 HPF 62.8% vs. mitosis ≤ 5/50 HPF 37.2% of patients).

The most common *KIT* exon 9 PV was the A502_Y503dup exon (8 out of 10 patients, 10% of all GISTs). These patients were mainly women (62.5%), with tumors frequently of small bowel origin (five out of eight tumors; 62.5%), a median mitotic rate of >5/50 HPF and a primitive tumor diameter of >5 cm in 75% of patients. The remainder of patients had *PDGFRA*-mutated GISTs, including two patients with D842V PV, and only three (3.8%) of these were *KIT/PDGFRA* WT GISTs.

Baseline clinical and pathological characteristics according to critical mutations are summarized in Table 2.

#### 2.2.2. Outcome Analysis and Objective Response in *KIT* Exon 11 Metastatic Patients

**Figure 2:**
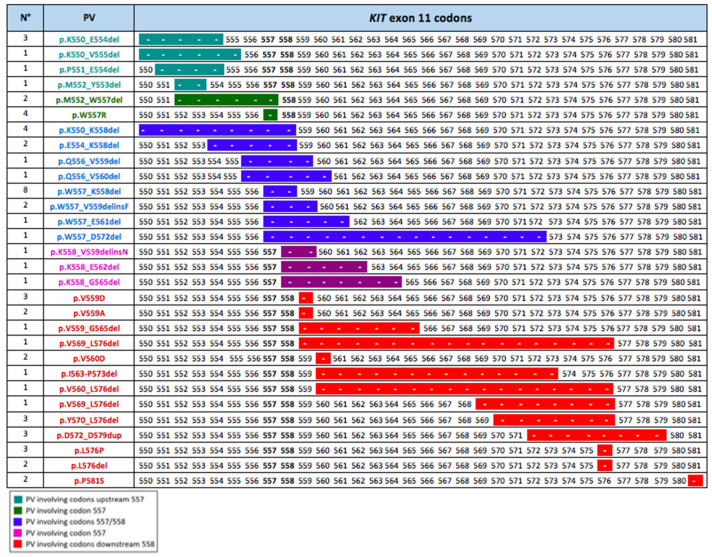
Codon location of the PVs in the population of metastatic *KIT* exon 11 patients treated with first-line imatinib. The different colors indicate the PVs positions as related to 557/558 critical codons: PVs involving codons upstream of codon 557; PVs involving codon 557; PVs involving codons 557 and 558; PVs involving codon 558; and PVs involving codons downstream of codon 558. The length of the line indicates the number of codons involved in each *KIT* exon 11 mutation.

We focused on the *KIT* exon 11 genotypic landscape because these are the most common molecular alterations in GISTs and, at the same time, they show wide variability in the duration of their response to imatinib. We classified the patients treated with first-line imatinib and *KIT* exon 11 mutation in two groups: (i) *KIT* Exon 11 deletion or insertion/deletion in codons 557 and/or 558 (named “D-557/8”); (ii) mutations other than those in D-557/8 (named “No-D-557/8”). Twenty-five (25) patients (41.7%) were in the D-557/8 Group, and 35 patients (58.3%) were in in the No-D-557/8 Group. The exact PV types and codon locations of the exon 11 study population are shown in Figure 2. 

**Figure 3:**
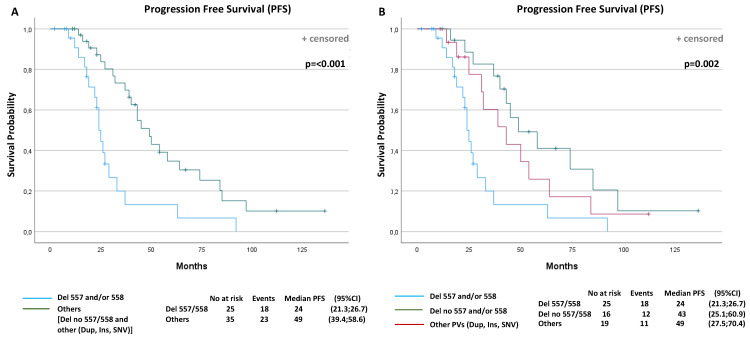

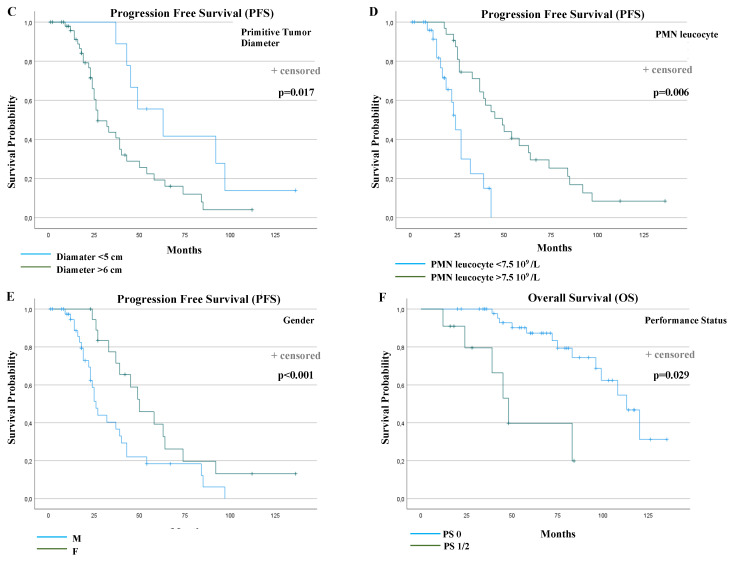
Progression-free survival (PFS) and overall survival (OS) curves according to prognostic factors that resulted statistically significantly from multivariable analyses. PFS according to: (**A**) *KIT* Exon 11 deletion and delins in codons 557/558 or other PV types (*p* < 0.001); (**B**) *KIT* Exon 11 deletion and delins in codons 557/558, deletion in other codons or other PV types (duplication, insertion and single nucleotide variant (SNV)) (*p* = 0.002); (**C**) primitive tumor diameter (*p* = 0.017); (**D**) polymorphonuclear (PMN) leucocyte (*p* = 0.006); (**E**) gender (*p* < 0.001); (**F**) OS according to performance status (*p* = 0.029).

A total of 41 events (progression or death) were observed (68.3%): 18 events in the D-557/8 group of 25 patients (72%) and 23 events in the No-D-557/8 group of 35 patients (65.7%). The overall median PFS was 37 months (95% confidence interval (CI): 26.8–47.1). The median PFS was 24 months (95% CI: 21.3, 26.7) for the D-557/8 Group and 49 months (95% CI: 39.4, 58.6) for the No-D-557/8 Group (*p* < 0.001) (Figure 3A).

Subsequently, in the next analysis, we have divided the group of other PVs than 557/558 deletions (NoD-557/8) into two further subgroups, to investigate the potential different impact on PFS: (i) patients with *KIT* Exon 11 deletion or delins in codons other than 557/558; (ii) patients with duplication, insertion or SNV of all codons. The median PFS was 43 months (95% CI: 25.1, 60.9) for the patients with deletion or delins in other codons than 557/558, and 49 months (95% CI: 27.5, 70.4) for patients with duplication, insertion or SNV (*p* = 0.002) (Figure 3B).

After a median follow-up of 107 months, 18 events (deaths) were observed (30%): 10 in 25 of D-557/8 patients (40%) and 8 in 35 (23%) of No-D-557/8 patients. The overall median OS was 102 months (95% CI: 89.8, 114.5). The median OS was 99 months (95% CI: 65.8, 132.2) for the exon 11 557/558 deletions or delins patients, and not reached (NR) for the group of patients with other mutations (*p* = 0.14).

Regarding the associations of *KIT* Exon 11 mutation type and best overall response (BOR) in metastatic GIST patients treated with first-line imatinib, patients with deletion or delins regardless of codon regions had a significantly better CR rate (557/558: 40%; other codons: 37.5%) compared to the patients with other PVs (Dup, Ins or SNV) (5.3%) (*p* = 0.006).

#### 2.2.3. Univariate and Multivariate Analysis

Table 3 summarizes the results of the univariable and multivariable prognostic factor analysis for PFS and overall survival (OS). Gender, performance status, the diameter of the primitive tumor, lymphopenia, polymorphonuclear (PMN) leucocytosis and *KIT* exon 11 PV type were found to be statistically significantly associated with PFS in univariable analyses. In the final multivariable Cox regression model, the following factors were significant: the female gender (median PFS: 26 versus 50 months, *p* < 0.001, HR: 0.23), a primitive tumor diameter ≤ 5 cm (median PFS: 27 versus 63 months, *p* = 0.017, HR: 3.39), a PMN leucocyte > 7.5 × 10^9^ (median PFS: 24 versus 49 months, *p* = 0.006, HR: 0.27) and the absence of *KIT* Exon 11 Del or Delins 557/558 (median PFS: 24 versus 45 months, *p* < 0.001, HR: 0.12). 

Regarding OS, performance status, lymphopenia and PMN leucocytosis were statistically significantly associated in univariable analyses. In the final multivariable model, only performance status remained statistically significant (median OS: 113 vs. 48 months, *p* = 0.029, HR: 3.97).

Therefore, these results showed that, in the population of metastatic *KIT* exon 11 mutated patients treated with first-line imatinib, the absence of *KIT* exon 11 deletions or delins 557/558, the female gender, a primitive tumor diameter ≤ 5 cm and polymorphonuclear leucocytosis > 7.5 × 10^9^/L were significant independent prognostic factors for longer PFS, while the performance status was the only significant prognostic factor for OS.

The PFS and OS curves were plotted according to each independent prognostic factor (Figure 3C–F).

## 3. Discussion

GIST oncogenic dependence on KIT and PDGFRA receptor is a paradigmatic model of oncogene addiction [22,23,24]. Imatinib is clinically efficacious in the treatment of advanced GIST patients, resulting in a median PFS of 20 months and a median overall survival (OS) of 57 months [14,25,26]. Although effective in the vast majority of patients, PFS to imatinib treatment may vary widely [27,28,29]. We know that in metastatic GIST patients, the most important predictive factors for response to imatinib are the *KIT* and *PDGFRA* genotypes [16,22]. We have learned that GIST patients harboring mutations in *KIT* exon 11 are highly sensitive to imatinib and show a deep and prolonged response. GISTs with *KIT* exon 9 mutation also benefit from imatinib, but are less sensitive to the standard dose of 400 mg/die and benefit from the increased dose of imatinib 800 mg/die [13,30]. The exon 11 mutations are the most common *KIT* mutations, and patients carrying these PVs represent a heterogeneous subgroup in terms of biological and clinical behavior, with 10% of patients remaining sensitive to imatinib despite the drug-selective pressure and keepingprogression-free after 10 years of treatment [16,31]. The lack of relevant predictive or prognostic clinical factors, according to the available data, makes the presence of this subset of long-term survivors of high biological and clinical interest. 

Based on the known predictive role of genotype, the variable clinical outcome in *KIT* exon 11 mutated patients could be related to intrinsic molecular features, such as a different PV type and the gene location of exon 11 *KIT* mutations. Previous findings on localized GIST provide the rationale to better investigate this hypothesis [18,19,20]. Indeed, several studies showed the prognostic significance of different types of *KIT* exon 11 mutations for recurrence-free survival in localized GIST patients treated with surgery [18]. *KIT* exon 11 deletions and deletions affecting *KIT* exon 11 codons 557 and/or 558 are associated with a poor prognosis and have been described as independent adverse prognostic factors for relapse [18,23]; conversely, other exon 11 *KIT* mutations, such as insertion and duplication mutations, are generally associated with a favorable outcome [18].

In terms of metastatic disease, previous studies have addressed the predictive role of *KIT* exon 11 PVs on response and survival to imatinib. However, to our knowledge, very few studies have further subdivided the *KIT* exon 11 mutated population-based on PVs. 

A first study compared the outcomes of patients with the most frequent deletion of *KIT* exon 11, the PV delWK557-558 and the deletion of Tyr568-570, which was selected because these tyrosines represent the first residues to be phosphorylated during activation and, thus, may be associated with specific effects on KIT signaling pathways. Metastatic GISTs, carrying one of two types of PVs (n.22 vs. n.14 patients, respectively) and treated with imatinib, showed no significant difference in terms of response rates, PFS and OS [23]. However, no other PVs were included in the analysis conducted for this study.

A subsequent study investigated the predictive role of *KIT* exon 11 mutations in a broader cohort of advanced patients, but with its main focus on PVs’ mutational locations. The researchers compared the molecular subgroups of advanced GIST treated in the BFR14 study of the French Sarcoma Group divided in terms of PV position: 557/558 codons, upstream or downstream exon 11 codons. Long-term outcome analysis showed that patients with alterations involving 557/558 codons developed secondary resistance more rapidly compared to the patients carrying the most distal exon 11 mutations (codon 559 or more) [21]. However, the role of a PV’s type, beyond its location, as a predictive biomarker for imatinib response has not been fully elucidated in metastatic patients.

Our study provides real-world evidence of the impact of type and location of *KIT* exon 11 PVs on clinical outcomes and response to therapy in advanced GIST patients treated with first-line imatinib 400. We show in a real-world population that the group of 557/558 mutations is not a homogeneous group in terms of response to imatinib: not all of the codons 557/558 PVs develop gain secondary resistance to imatinib more rapidly than other *KIT* PVs, as a markedly poorer PFS curve is shown for deletions involving codons 557/558. This result reaches an independent value in the multivariate model. Patients with deletion or insertion/deletion in codons 557/558 had a median PFS of 24 months; this PFS is in line with literature data in the metastatic setting [14], but is shorter compared to patients with a deletion in other codons (median PFS: 43 months) or patients with duplication, insertion or SNV (median PFS: 49 months) (p = 0.002). Multivariable prognostic factor analysis performed to assess the impact of the different baseline covariates on PFS and OS, showed that the absence of *KIT* Exon 11 del or delins 557/558, the female gender, a primitive tumor diameter ≤5 cm and PMN leucocytosis (>7.5 × 10^9^/L) were significant prognostic factors for longer PFS to first-line imatinib; only performance status remains statistically significant in the multivariate model for OS.

These data, combined with previous findings in patients with localized diseases, confirm an aggressive biology of 557/558 exon 11 deletions or delins even in the metastatic setting, particularly for specific PVs such as the *KIT* p.W557_K558del, which explains their high prevalence in advanced GISTs [32]. The shorter PFS associated with 557/558 deletions could be biologically explained by their specifically designated role in the process of tyrosine kinase activation of the different segments of the KIT receptor. It is known from previous studies that the oncogenic KIT signaling mechanisms varied depending on the exact type and location of the PVs [33,34,35]. The 557/558 codon region contains residues that exert a critical autoinhibitory role on kinase activity, repressing the ligand-independent activation. The exon 11 codons 557/558 deletions may perturb KIT kinase autoinhibition by controlling the a-helical conformation, essentially leading to increased spontaneous receptor phosphorylation, the activation of Ras/Raf/MAPK, JAK/STAT3 and PI3K/Akt/mTOR downstream signaling pathways, ultimately leading tumor cell proliferation and the inhibition of apoptosis [36].

To our knowledge and based on the best data available today, this is the study that has provided the most representative picture of the predictive role of *KIT* exon 11 pathogenic variants in metastatic patients treated with first-line imatinib. It remains to be elucidated whether other convincing factors (such as microenvironment immunological characteristics or clinical factors not yet investigated) may have a further relevant impact on tumor response and progression-free survival.

## 4. Patients and Methods

### 4.1. Study Population

In the current study, clinicopathological variables and mutational status data were analyzed from a GIST System database prospectively collected in an Italian referral center for diagnosis and treatment of soft tissue sarcoma and GIST: the “Sicilian Regional Center for the Prevention, Diagnosis, and Treatment of Rare and Heredo–Familial Tumors” of the Section of Medical Oncology of University Hospital Policlinico “P. Giaccone” of Palermo. In the study the patients afferent to the center between January 2005 and September 2020 were included, based on retrievable tumor specimens in order to centrally repeat mutational analysis. The metastatic GIST patients treated with first-line imatinib 400 therapy were included in the analyses of the predictive role of mutation type and codon location. The information collected from these patients included gender, age, site of the origin of primary tumors, primitive tumor diameter and mitosis, *KIT* and *PDGFRA* pathogenic variant (PV) classification, data on active disease sites, best overall response (BOR) to imatinib (progression disease (PD), stable disease (SD), partial response (PR), complete response (CR)) assessed according to response evaluation criteria in solid tumors (RECIST version 1.1.), progression-free survival (PFS) to imatinib treatment and overall survival (OS). The association between *KIT* Exon 11 mutation type and BOR, PFS and OS was evaluated. The clinical, pathological and genetic information were anonymously recorded for all patients who previously provided written informed consent. This study was conducted in accordance with the Declaration of Helsinki and Good Clinical Practice guidelines.

### 4.2. Mutation Analysis

The diagnosis of GIST was made based on histopathologic assessment and immunohistochemical staining for CD117 antigen expression from local pathology testing of diagnostic core biopsies or tumor resections for clinical use. The pathologists, with special interest in sarcoma pathology, also reported tumor mitoses from 50 HPFs and diameter lesions. Four (4)-μm thick FFPE tissue sections were deparaffinized with a Deparaffinization Solution (Qiagen, Hilden, Germany) and genomic DNA was extracted using a QIAamp DNA FFPE Tissue Kit (Qiagen, Hilden, Germany) according to the manufacturer’s instructions. Extracted DNA was quantified using a Qubit^®^ 3.0 fluorometer (Thermofisher Scientific, Waltham, MA, USA) and its quality was assessed by using a SeqStudio Genetic Analyzer (Applied Biosystem). All GISTs were centrally examined for somatic mutations in *KIT* (exons 9, 11, 13 and 17) and *PDGFRA* (exons 12, 14 and 18) by polymerase chain reaction (PCR) amplification and direct Sanger sequencing. PCR reactions were run on Veriti Thermal Cycler (Applide Biosystem) and Sanger sequencing wasperformed using a BigDye Terminator 3.1 Cycle Sequencing Kit (Life Technologies, Carlsbad, CA, USA) on the SeqStudio Genetic Analyzer (Applied Biosystem, Foster City, CA, USA), according to manufacturer’s protocols. PVs were confirmed using Sanger sequencing with a BigDye Terminator 3.1 Cycle Sequencing Kit (Life Technologies) on the SeqStudio Genetic Analyzer (Applied Biosystem). Samples that scored negative were further profiled using a targeted next generation sequencing (NGS) panel for the presence of hot spot mutations in H/K/N RAS, BRAF and NTRK using an Ion Torrent S5 (Thermofisher Scientific) instrument. For all detected PVs found, a gene name, a nucleotide change (c.notation) and an amino acid change (p.notation) were codified. The classification of the variant was performed after consulting the databases “Catalogue Of Somatic Mutations In Cancer” (COSMIC) and ClinVar. According to the aim of the study, only the *KIT* and *PDGFRA* PVs were included in the following analyses.

### 4.3. Statistical Analysis

PFS was calculated from beginning of the imatinib treatment to death by any cause, disease progression or last follow-up (censored patients). OS was calculated from the beginning of the imatinib treatment to death by any cause or last follow-up (censored patients). The analyses of PFS and OS between groups were compared using the Kaplan–Meier method and a log-rank test. To identify independent prognostic factors for PFS and OS, univariate and multivariate Cox proportional hazard regression models were built. All tests were performed with a significance level of *p* = 0.05. Statistical analyses were conducted using IBM SPSS Statistics for Windows Version 25.0 (IBM Corporation, Armonk, NY, USA).

## 5. Conclusions

From previous studies on GIST, we have learned that, in metastatic patients, the main predictive factor for the duration of response to imatinib is the genotype [13]. In the *KIT* exon 11 mutated patients, who are the most common molecular-based subgroup of advanced GIST patients, our results reveal the important impact of the PVs’ type and location on PFS. We detected a significantly shorter PFS for those patients carrying deletions involving 557/558 codons compared to the patients with deletions in other codons or patients with duplications, insertions or SNVs. These data, combined with the existing evidence on the negative role of 557/558 deletion for RFS in localized patients treated with surgery [18], confirm the aggressive biology of 557/558 exon 11 deletions or delins in the metastatic setting as well. These findings have potential clinical utility because they only allow an understanding of why a subgroup of patients responds to imatinib better than other groups, but also predict at baseline which GIST patients would experience resistance to treatment earlier. This study also supports the use of stratification by mutational type and codon location in future clinical trials.

## Figures and Tables

**Table 1:** Number of GIST patients with known *KIT* and *PDGFRA* mutational status. The description is based on affected gene and exon, mutation type (deletion, deletion/insertion, duplication, insertion and single nucleotide variant) and exact *KIT* or *PDGFRA* pathogenic variant.

Gene	Exon	Mutation Type	Number of Patients (%)		Pathogenic Variant	N.
***KIT*** **n. 174 (84.5%)**	**Exon 9** **20 (9.7%)**	Duplication	15 (7.3%)		p.A502_Y503dup	15
		Insertion	3 (1.5%)		p.Y503_F504insAY	3
		SNV	2 (1%)		p.S464L	2
	**Exon 11** **154 (74.8%)**	Deletion	95 (46.1%)	Codons 557–558 60 (29.1%)	p.W557_K558del	16
					p.K550_K558del	6
					p.E554_K558del	5
					Others *	33
				Upstream codon 557 18 (8.7%)	p.K550_E554del	6
					p.M552_Y553del	3
					p.K550_V555del	2
					Others *	7
				Downstream codon 558 17 (8.2%)	Involving codons 568–570: p.V559_T574del p.V560_L576del p.I563-P573del p.N567_P573del	1 1 1 1
					Other codons: p.V560del p.L576del p.Y570_L576del Others	3 3 2 5
		Deletion/Insertion	9 (4.4%)		p.K558_V559delinsN	3
					p.W557_V559delinsF	3
					p.K558_G565delinsR	2
					p.W557_E561delinsPS	1
		Insertion	2 (1%)		p.K558_V559insS	2
		Duplication	10 (4.8%)		p.D572_D579dup	9
					p.D572_P585dup	1
		SNV	38 (18.4%)		p.W557R	10
					p.V559D	6
					p.V560D	6
					p.L576P	5
					Others	11
***PDGFRA*** **n. 14** **(6.8%)**	**Exon 12** **3 (1.5%)**	Deletion	1 (0.5%)		p.W559_R560del	1
		SNV	2 (1%)		p.P581S	2
	**Exon 18** **11 (5.3%)**	Deletion	1 (0.5%)		p.D842_M844del	1
		Deletion/Insertion	1 (0.5%)		p.D842_I843delinsV	1
		SNV	5 (1.9%)		p.D842V	5
		Others	4 (1.9%)			4
***KIT/PDGFRA*** **Wild Type** **n. 18 (8.7%)**	-		-		-	-

* Others: PVs with numbers < 2. SNV: single nucleotide variant.

**Table 2:** The clinical and pathological characteristics of metastatic GIST patients, according to the type and location of the critical mutations. The patients were classified based on genotyping as follows: (i) *KIT* exon 11 deletion or deletion/insertion involving 557 and/or 558 codons versus other *KIT* exon 11 PVs; (ii) *KIT* exon 9 A502-Y503 codon duplication versus other *KIT* exon 9 PVs; and (iii) the *PDGFRA* D842V single nucleotide variant versus other *PDGFRA* PVs.

Characteristic	N. of Patients (%)	*KIT*				*PDGFRA*		*KIT/* *PDGFRA* WT
		Exon 11 *		Exon 9		Exons 12, 14, 18		-
		Del/ Delins Codons 557/558	Others ^1^	p.A502_Y503dup	Others ^2^	no-D842V	D842V	-
N. of patients (%)	80	25 (31.3)	35 (43.7)	8 (10)	2 (2.5)	5 (6.2)	2 (2.5)	3 (3.8)
Sex								
Male Female	50 (62.5) 30 (37.5)	16 (64) 9 (36)	25 (71.4) 10 (28.6)	3 (37.5) 5 (62.5)	2 (100) 0 (0)	2 (40) 3 (60)	1 (50) 1 (50)	1 (33.3) 2 (66.7)
Age at diagnosis (y):								
Median Range	57 30–81	57 45–74	57 31–81	59 49–77	37 30–44	50 36–59	57.5 36–79	50 50–58
Site of origin								
Gastric Small bowel Other GI	27 (33.8) 34 (42.5) 19 (23.7)	15 (60) 7 (28) 3 (12)	8 (22.8) 18 (51.5) 9 (25.7)	0 (0) 5 (62.5) 3 (37.5)	0 (0) 1 (50) 1 (50)	1 (20) 2 (40) 2 (40)	1 (50) 1 (50) 0 (0)	2 (66.7) 0 (0) 1 (33.3)
Primitive tumor diameter								
≤5 cm >5 cm	14 (17.5) 66 (82.5)	2 (8) 23 (92)	8 (22.8) 27 (77.2)	2 (25) 6 (75)	0 (0) 2 (100)	2 (40) 3 (60)	0 (0) 2 (100)	0 (0) 3 (100)
Mitosis								
≤5/50 HPF >5/50 HPF	25 (31.3) 55 (68.7)	6 (24) 19 (76)	13 (37.2) 22 (62.8)	2 (25) 6 (75)	0 (0) 2 (100)	3 (60) 2 (40)	1 (50) 1 (50)	0 (0) 3 (100)
Site of metastases								
Liver Peritoneum Liver and peritoneum	31 (38.7) 31 (38.7) 18 (22.6)	10 (40) 11 (44) 4 (16)	15 (42.9) 13 (37.1) 7 (20)	2 (25) 4 (50) 2 (25)	0 (0) 2 (100) 0 (0)	2 (40) 1 (20) 2 (40)	0 (0) 0 (0) 2 (100)	2 (66.7) 0 (0) 1 (33.3)

SNV: single nucleotide variant; WT: wild type. * Comparison exon 11 del or delins 557 and/or 558 versus other exon 11 mutations; ^1^ Other mutations than deletion or deletion/insertion codons 557 and/or 558; ^2^ Others mutations include one insertion (p.Y503_F504insAY) and one SNV (p.S464L).

**Table 3:** Univariate and multivariate analysis of prognostic factors of PFS and OS in GIST exon 11 mutated patients treated with first-line imatinib. Gender, performance status, primitive tumor diameter, number of mitoses, liver and peritoneal metastatic involvement, lymphopenia, polymorphonuclear leucocytosis and *KIT* exon 11 codon 557 and/or 558 deletion or deletion/insertion were evaluated in the Cox regression model.

PFS	Univariate Cox Regression		Multivariable Cox Regression	
	HR (95%CI)	*p*-Value	HR (95%CI)	*p*-Value
Gender (F vs. M)	0.46 (0.24–0.89)	0.022	0.23 (0.09–0.53)	<0.001
Performance status (PS) (1/2 vs. 0)	3.64 (1.47–9.03)	0.005	3.13 (0.89–10.98)	0.073
Primitive tumor diameter (≤5 cm vs. >5 cm)	2.62 (1.13–6.07)	0.025	3.39 (1.25–9.22)	0.017
Mitosis (≤5/50 HPF vs. >5/50 HPF)	1.14 (0.59–2.21)	NS		
Gastric site of origin (No vs. yes)	1.27 (0.58–2.81)	NS		
Liver and peritoneal involvement (No vs. yes)	0.83 (0.32–2.14)	NS		
Lymphopenia (≤1 vs. <1 G/L)	2.11 (1.01–4.42)	0.046	1.05 (0.35–3.19)	0.92
Polymorphonuclear leucocyte (>7.5 × 10^9^ vs. ≤7.5 × 10^9^/L)	0.24 (0.11–0.51)	<0.001	0.27 (0.10–0.68)	0.006
Exon 11 Del or Delins 557 and/or 558 (No vs. yes)	0.36 (0.19–0.69)	0.002	0.12 (0.04–0.31)	<0.001
**OS**	**Univariate Cox Regression**		**Multivariable Cox Regression**	
	**HR (95%CI)**	***p*-value**	**HR (95%CI)**	***p*-value**
Gender (F vs. M)	1.19 (0.48–2.98)	NS		
Performance status (PS) (1/2 vs. 0)	6.35 (2.17–18.58)	<0.001	3.97 (1.15–13.68)	0.029
Primitive Tumor diameter (≤5 cm vs. >5 cm)	2.17 (0.60–7.73)	NS		
Mitosis (≤5/50 HPF vs. >5/50 HPF)	1.39 (0.50–3.83)	NS		
Gastric site of origin (No vs. yes)	0.40 (0.13–1.21)	NS		
Liver and peritoneal involvement (No vs. yes)	2.38 (0.63–9.07)	NS		
Lymphopenia (≤1 vs. <1 G/L)	3.54 (1.35–9.27)	0.01	2.16 (0.71–6.55)	NS
Polymorphonuclear leucocyte (>7.5 × 10^9^ vs. ≤7.5 × 10^9^/L)	0.38 (0.15–9.81)	0.04	0.76 (0.25–2.38)	NS
Exon 11 Del or Delins 557 and/or 558 (No vs. yes)	0.49 (0.20–1.24)	NS		

CI: confidence interval; Del: deletions; Delins: deletions/insertions; HR: hazard ratio; OS: overall survival; and PFS: progression free survival.

## Data Availability

No new data were created or analyzed in this study. Data sharing is not applicable to this article.
